# University managers or institutional leaders? An exploration of top-level leadership in Chinese universities

**DOI:** 10.1007/s10734-023-01031-x

**Published:** 2023-04-10

**Authors:** Jieyu Ruan, Yuzhuo Cai, Bjørn Stensaker

**Affiliations:** 1 Faculty of Social Sciences, Nord University, 8049 Bodø, Norway; 2 Faculty of Management and Business, Tampere University, 33014 Tampere, Finland; 3 Faculty of Educational Sciences, University of Oslo, 0371 Oslo, Norway

**Keywords:** Institutional leadership, Management, Dual governance structure, Chinese higher education

## Abstract

In China, higher education institutions (HEIs) have a governance arrangement in which the university president and the party secretary occupy key roles. However, their legal roles as institutional leaders are vaguely specified in existing legal frameworks. Based on a four-dimensional theoretical model, this paper (i) clarifies the leadership roles in the dual governance structure, (ii) explores how HEI leaders (i.e. presidents and party secretaries) perceive their leadership, and (iii) applies the unique Chinese practices as a valuable test bed for critical reflections on how existing theoretical models of leadership are relevant in Chinese contexts. Through in-depth interviews with six top-level leaders from six Chinese public HEIs, our findings indicate that Chinese HEI leaders apply more structural than symbolic dimensions in their leadership practices. Whereas studies on institutional leadership conducted outside China tend to highlight the symbolic dimensions of leadership practices, our study suggests that top-level Chinese HEI leaders may assume the role of university managers rather than institutional leaders. We offer some reflections on the relevance of existing theoretical models of leadership and suggest the directions for further theoretical enhancement.

**Supplementary Information:**

The online version contains supplementary material available at 10.1007/s10734-023-01031-x.

## Introduction

Global studies of organisation and management in higher education appear to be facing a dilemma. On the one hand, the theories and concepts that underpin many studies often originate from Western contexts (Cai & Mehari, 2015). On the other hand, researchers seem to struggle to understand some intriguing and complicated phenomena in non-Western contexts due to a lack of relevant theoretical lenses (Huang, 2017). Hence, there is a need to find ways to align theories with the diverse empirical realities of current studies (Welch & Wong, 1998).

Although there is considerable theoretical richness in the area (Lowe & Gardner, 2000), cognitive models of academic leadership have been a dominant perspective for the past four decades (e.g. Baker et al., 2020; Ngayo Fotso, 2021). The cognitive approach investigates how leaders make sense of a complicated and fluid world by assessing organisational needs and circumstances as well as relating results to causes. In short, leaders interpret situations through different cognitive frames before deciding on a course of action (Birnbaum, 1988; Bolman & Deal, 2013; Kezar et al., 2006). Chinese higher education, with its unique social and cultural contexts and organisational structure (Marginson & Yang, 2022), can serve as a valuable test bed for critical reflections on how well-established Western theories apply to the Chinese context.

Our point of departure for this inquiry concerns a unique governance arrangement in Chinese universities, where the university president and the party secretary occupy vital roles—a dual form of leadership that is quite unique to China. Our paper responds to two research challenges: one concerns the analytical interpretation of China’s complex university leadership, while the other addresses the theoretical foundations that may help us shed light on this complex institutional governance arrangement. By selecting one well-established cognitive leadership model—Bolman and Deal’s four-dimensional leadership framework (2013)—as an analytical tool, our article is guided by the following research questions:

- How do top-level leaders in Chinese HEIs perceive their leadership?
- How can their leadership perceptions inform our understanding of the dual governance arrangement in Chinese public HEIs?
- How relevant is Bolman and Deal’s framework in the Chinese context?

## Dual leadership in Chinese higher education: a literature review

At the system level, higher education in China is predominantly public, as the vast majority of students are enrolled in public universities (Cai & Yan, 2017). Chinese public HEIs are guided and administrated by the State Council and local governments at the provincial or municipal levels (Liu & Wang, 2019). Since the 1990s, China has implemented profound reforms in higher education governance, largely influenced by global reform ideologies/trends (Cai, 2010). Nevertheless, essential powers remain in the hands of the state, such as controlling ideo-political education and appointing presidents and party secretaries (Han & Xu, 2019; Li & Yang, 2014). This situation is depicted as ‘semi-independence’ (Li & Yang, 2014) or, more vividly, as ‘dancing in a cage’ (Yang et al., 2007). Both terms imply that the influence of global ideas on higher education governance (mainly from the West), endorsing institutional autonomy, has been counteracted by China’s unique political system and cultural tradition (Jiang & Mok, 2019; Zha & Shen, 2018). Such ideological paradoxes are legitimised by the phrase ‘Higher Education with Chinese Characteristics’, which is widely used in China (Ma & Cai, 2021).

Within Chinese public HEIs, a unique feature of the governance arrangement is the existence of a party secretary as an integrated part of the top-level leadership alongside a university president (Liu & Wang, 2019), with an aim to ensure the infusion of socialism with Chinese characteristics throughout the higher education system (Li & Zhu, 2019). This dual governance arrangement has long been practised in Chinese higher education, and it was formally regulated by the Chinese Higher Education Law. According to the law, ‘in higher education institutions run by the State, the system shall be applied under which the presidents take overall responsibility under the leadership of the primary committees of the Communist Party of China (PCCPC) in HEIs’ (Article 39). It is abbreviated as Presidential Responsibility under the Leadership of the Party Committee (PRLPC). Despite the existence of the Higher Education Law, it is still difficult to understand the roles of the primary committee and the president in the daily operation of their HEIs (Huang, 2017) and how responsibility and decision-making are distributed between the party secretary, who heads the primary committee, and the president (Jiang & Li, 2016). This uncharted realm of dual governance creates the potential for both collaboration and conflict among top-level leaders.

However, there is little research on implementing dual governance in Chinese higher education. Since no detailed account of the two groups of institutional leaders was available, Huang (2017) employed elite dualism theory (Zang, 2003) to analyse the personal attributes of Chinese HEI leaders found on websites and argued that dualism is a form of collective leadership. According to Wang (2010), the distinction between political leadership and administrative management has blurred in practice, since political control is built into the administrative structure.

In addition, few researchers have paid attention to senior leadership teams. Unlike in Western higher education, top leaders in Chinese public HEIs see themselves as government officials, as they are appointed and dismissed by either central or local authorities (Wang, 2010). To select candidates for top-level higher education leaders, the government uses criteria similar to those used for government officials. Once appointed, these institutional leaders are entitled to administrative titles corresponding to their HEIs (Wu, 2006), and presidents have the same administrative status as party secretaries (Liu, 2017). However, according to Huang (2017, p. 79), ‘it seems that different criteria are utilised in selecting and appointing party leaders and administrative leaders, derived from differences in their roles and responsibilities’. Some research has shown that party secretaries are more likely to be administrative leaders, whereas presidents are more likely to be academic leaders (Jiang & Li, 2012; Jiang et al., 2008). They may also have different beliefs about institutional governance: whereas party secretaries tend to put a greater emphasis on political construction and the development of relationships between university and society, presidents are generally more concerned with specific issues of university development (Ling & Xu, 2020). Moreover, they may demonstrate different leadership behaviours due to their different career backgrounds and networks in society (Ma & Cai, 2021).

Despite the differences mentioned above between party and administrative leaders, confusion about the lines of accountability and responsibility could arise in the dual governance arrangement. For example, the party members of the Council of University Presidents (CUP) are usually members of the Communist Party of China’s University Committee (CPCUC). This overlap merges academic and political management roles (Liu, 2017). Notably, a dual leadership position is not unusual. The percentage of presidents assuming (vice-) party secretaries was 22.5% in Project 211 universities (Li, 2016), 60% in HEIs governed by the Beijing municipal government (Li, 2019), and 91% in 75 HEIs governed by the Ministry of Education. We calculated this latest data using information from the website (MoE, 2022). Moreover, there appear to be no clear lines of demarcation between administration and politics in the career advancement of top leaders. Party secretaries who used to be (vice-) presidents were found in all eight types of Chinese HEIs, albeit in various proportions (Jiang et al., 2008, p. 60). In such a dual governance system, there appears to be a need for clarification of the top leadership team’s roles and the relationship between the president and the party secretary (Li & Yang, 2014; Liu, 2017). In sum, the literature lacks deep analyses of Chinese top leaders’ perceptions of leadership and management, especially from a theoretical perspective.

## Theoretical framework

Although leadership and management are somewhat overlapping concepts, there is a longstanding debate over the relationship between them. Management is mainly concerned with organisational structure and elements—planning, organising, executing, and controlling—while leadership is oriented towards change and long-term thinking, visioning, networking, establishing relationships, and going beyond immediate formal jurisdictions (Bolman & Deal, 2013, p. 345). Similarly, in educational organisations, management deals with responsibility, implementation, proper function, and approaches to attaining organisational goals, whereas leadership engages with values, purpose, and influence (Bush, 2020; Connolly et al., 2019). However, differentiating management from leadership can be problematic. Modern organisations require both management and leadership (Bolman & Deal, 2013, p. viii). Managers and leaders can also take on mixed roles, where some managers become leaders and vice versa (Hoff, 1999).

**Fig. 1:**
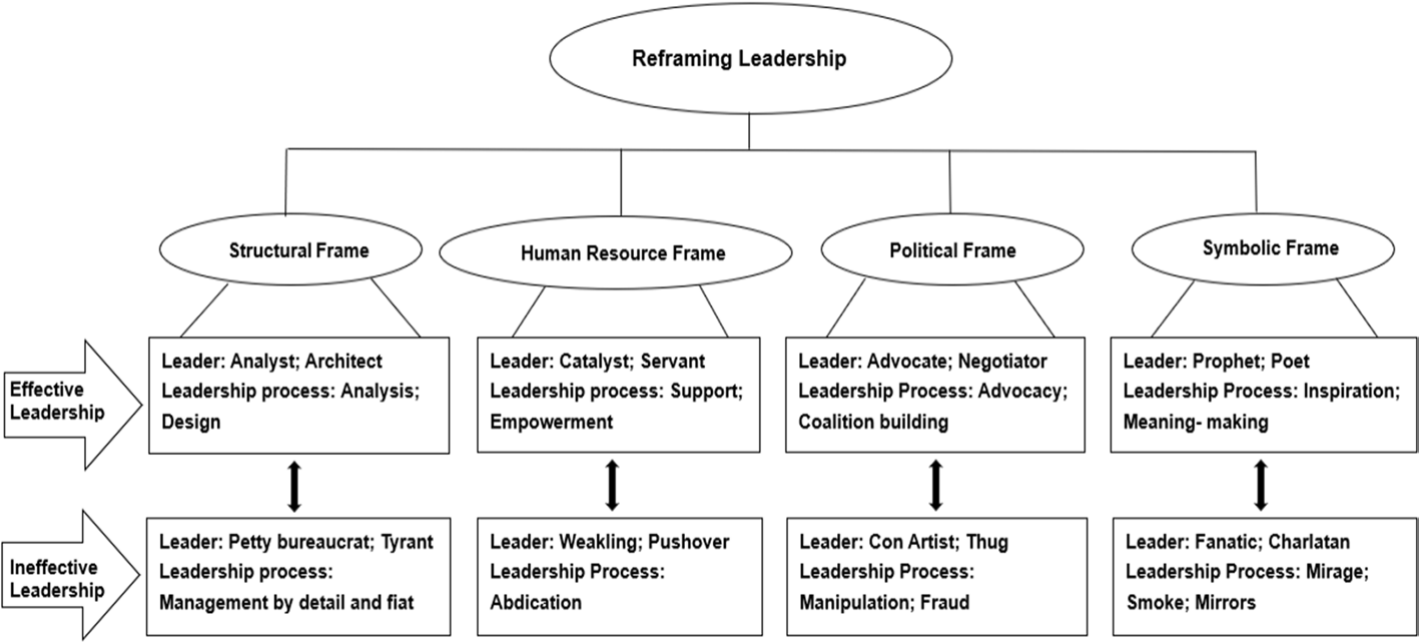
Bolman and Deal’s four-frame leadership model. Source: Adapted from Bolman and Deal (2013, p. 355)

Cognitive theoretical frameworks often offer conceptualisations of the diverse dimensions that could shape leadership attention and behaviour, including political, collegial, anarchic, bureaucratic, and symbolic frames of understanding (Birnbaum, 1988; Bolman & Deal, 2013; Kezar et al., 2006). Bolman and Deal take a position on the dichotomy between leadership and management, which was a major consideration when selecting their four-frame model for our empirical analysis. Bolman and Deal (2013) developed four scenarios or dimensions (Fig. 1) to reframe leadership and link leadership studies closer to organization studies. As shown in Fig. 1, the four dimensions represent distinct ways in ‘which leaders see their organisations, the ways in which they think about the strategic actions they should take and the implicit models of leadership that influence how they enact their roles’ (Birnbaum, 1992, p. 23).

In the structural frame, leaders prioritize the clarification of goals, the alignment of organizational structure with its environment, and the establishment of appropriate roles and relationships for the task at hand. In an effective organisation, policies, linkages, and scopes of authority are well defined and generally recognised. People are aware of their obligations and contributions because a rational structure has been established. The human resource frame focuses on employees and assumes that an effective organisation is one where staff members are committed and loyal to the organisation and in which their personal needs and goals are met. The political frame understands the organisation as an arena for interest struggles. An effective organisation recognises political realities and solves or copes with disputes. In this frame, leadership is about building a power base and wielding power properly while demonstrating sensitivity. The symbolic frame is about creating meaning and identifying factors that provide staff with direction and purpose. Effective symbolic leaders are enthusiastic about making their organisation unique and conveying their passion to people. The symbolic frame emphasises slogans, stories, rallies, ceremonies, and awards as sense-making instruments for developing a shared culture (Bolman & Deal, 2013). Under each frame, Bolman and Deal present leadership roles and processes that connect to effective and ineffective leadership. Each frame emphasises significant possibilities for leadership, but each one, by itself, is incomplete. Ideally, leaders combine multiple frames into a comprehensive leadership strategy (Bolman & Deal, 2013), as ‘an increasingly complex and turbulent organizational world demands greater cognitive complexity’ (Bolman & Deal, 1991, p. 528). Combining different frames yields the most effective leadership style (Thompson, 2000, p. 970).

More importantly, Bolman and Deal link the four frames to the distinction between managerial and leadership effectiveness. Managerial effectiveness is most closely associated with a structural orientation, whereas the symbolic and political frames are the two best predictors of leadership effectiveness (Bolman & Deal, 1991). The human resource frame can be related to both managerial and leadership effectiveness. Accordingly, organisational heads who prefer to think and act from the structural frame are more likely to maintain the status quo in their organisations and thus play the role of managers. By contrast, those who prefer to think and act from the symbolic and political frames tend to bring changes to their organisations, thereby playing the role of leaders. The distinction between leadership and management effectiveness served as an overarching guideline for our analysis, as it could provide a clear benchmark for clarifying leadership roles, making it easier to see which group of leaders is more management- or leadership-oriented.

As a theoretical framework in leadership studies, Bolman and Deal’s four-frame model has been used to study leaders in different leadership positions in various organisations (Bolman, 2022), including higher education (Vuori, 2018). In academic leadership studies, it has been used to evaluate leadership effectiveness among programme leaders in American HEIs (Phillips & Baron, 2013), determine the perceptual congruence of leadership orientations in Malaysia (Joo et al., 2014; Tan et al., 2015) and different US states (Bensimon, 1990), and identify the connection between chairpersons’ leadership orientations and subordinates’ organisational commitments in Iranian and Indian HEIs (Shirbagi, 2007). The model is also helpful in comparing differences in leadership between national contexts. For example, in American universities, the human resource frame was found to be the dominant leadership orientation, followed by the structural frame (Beck-Frazier et al., 2007, p. 101; Sypawka et al., 2010, p. 67), with the political frame being employed the least (Maitra, 2007, p. 101; Welch, 2002, p. 105). In job advertisements for university presidents in Canada, more emphasis is placed on their symbolic roles than on their human resource roles (Lavigne & Sá, 2021). A key finding in these studies is that the symbolic frame is becoming increasingly visible in leadership orientations across national contexts (Lavigne & Sá, 2021; Welch, 2002, p. 105).

Last but not least, the four-frame model’s composition approach suggests that it has the potential for theoretical generalisation by absorbing more empirical information more than being designed for empirical generalisation. Rather than being regarded as a theory, it is built on the integration of some central schools of organisational theory—taking different ideas from rationalist systems theories, the human resource school, the political school, and the symbolic school (Vuori, 2018). Thus, the framework contains a range of theoretical contributions that could be relevant for explaining and understanding diverse empirical settings, including the Chinese one.

## Research methods

We adopted a qualitative research design, following the suggestion that qualitative research is best suited to exploring unknown social phenomena (Creswell, 2014) and gaining a deep understanding of human behaviour and the reasons behind it (Bryman, 2012). This was the case in our study, since HEI leaders’ perceptions of leadership in Chinese higher education is uncharted territory.

The data were mainly obtained from six semi-structured interviews. To have a stable point of departure in a highly diversified higher education system (Dong et al., 2020), we used the criterion of ‘incumbent top-level leaders in public HEIs’ as a rough guide for sample selection. Although several institutional types can be distinguished among the nearly 3000 Chinese public HEIs, what they have in common is the university governance model (Cai & Yan, 2017), which in turn affects institutional management and leadership (Liu & Wang, 2019). The potential participants thus included incumbent university presidents, party secretaries, university vice-presidents, and party vice-secretaries. Regarding the sampling strategy, we initially pursued a gender balance and tried to approach leaders who did not simultaneously hold political and administrative leadership positions, since this would allow us to better investigate whether the dual governance tracks interact. Given that male university leaders heavily outnumbered female university leaders in China, we began by tentatively contacting male leaders. Unfortunately, nobody responded. Given the difficulty of reaching top-level university leaders in China, we applied a pragmatic strategy: purposive sampling was carried out using convenience and snowball sampling. We approached potential interviewees through an international conference, and some academic leaders helped recruit additional participants through personal connections. In total, we completed six interviews in 2020. All the interviewees were female. This was an unintended consequence of the challenges and uncertainties associated with finding Chinese higher education leaders who were willing to participate.

Basic information about the six participants is presented in Table 1. None of them had dual leadership positions except for the president, UP1, who also served as party vice-secretary. Nonetheless, the interviewees’ career profiles showed that a party leader was once a top-level administrative leader, and an administrative leader had previously served as a top-level party leader. Two of the six HEIs are part of the Double First-Class Initiative; one is governed by a central government agency, two by provincial governments, and one by a municipal government.

**Table 1:** Basic information about the participants

Participant code	Leadership position	HEI code	Interview language
UP1	University president	U1	Chinese
UP2	University vice-president	U2	Chinese
UP3	University vice-president	U3	Chinese
PS1	Party secretary	P1	Chinese
PS2	Party vice-secretary	P2	Chinese
PS3	Party vice-secretary	P3	Chinese

Each of the six interviews lasted an average of one hour and 20 min. Each interview was completed at once. Due to the COVID-19 outbreak, interviews were conducted via WeChat. The interviewees were asked to describe their leadership experiences and principles, self-reported leadership styles and influences, and their opinions on leadership roles and responsibilities as well as university governance and management. As this was an exploratory study, our interview questions evolved along with the process. After completing an interview, we tried to learn lessons and improve the interview questions for the next one. As a result, each interview constituted a pilot for the next one. We supplemented the interview guide as the Appendix 1 to our article. Since we only published the findings on leadership and governance, we merely presented the interview questions relevant to the data reported. This means that the participants answered more questions than those in the pre-designed interview questions (Appendix 1). To increase the validity of the research findings (Bryman, 2012), we used policy documents issued in the last decade as secondary data sources to triangulate the interview data in the political frame.

We used NVivo 12 to code the interview transcripts, which constituted the major body of data. Bolman and Deal (1991) provided the Criteria for Coding Frame Responses in their study. We employed this coding structure as the initial template for the data analysis. Template analysis, as a form of thematic analysis, ‘balances a relatively high degree of structure in the process of analysing textual data with the flexibility to adapt it to the needs of a particular study’ (King, 2012, p. 426). The four prior themes—structure frame, human resource frame, political frame, and symbolic frame—had previously been defined and aided in our initial coding phase. They were also so well established in previous studies that we highly expected them to appear in this study’s data. Nonetheless, we developed and categorised the codes based on the data collected in the Chinese empirical setting. Thus, we constantly checked whether there were any codes outside the established themes and if any new codes or themes emerged. The coding table generated from this study is presented in Appendix 2.

## Empirical analysis

### Leadership frames identified

Guided by Bolman and Deal’s coding structure, all four frames were identified in the interview analysis (Appendix 2). This indicates that Chinese institutional leaders viewed their HEIs and leadership through a variety of cognitive lenses.

The codes in the structural frame suggest that organisational control systems and formal rules governed institutional leaders’ daily work, and they tried to ensure that their HEIs operated as ‘mechanistic hierarchies with clearly established lines of authority’ (Bensimon, 1989). They exerted power to set institutional goals and priorities (UP1, UP2, and PS1); brought changes to their HEIs by constructing and restructuring organisational units (UP1, UP2, UP3, PS1, and PS3) as well as encouraging reform and innovation (PS3); assigned people and tasks in terms of positional hierarchy and scope of authority (UP1 and PS1) and supervised the implementation process (PS1); launched the performance evaluation processes and pinpointed the criteria for punishment and reward (UP2 and UP3); made great efforts to promote institutionalisation by adhering to rules and regulations, studying government policies and drafting institutional rules according to them (UP2, PS1, and PS2); and used legal means to solve problems (PS1). They believed institutionalisation was important for today’s universities (UP2, PS2, and PS3) and thus valued policies, rules, principles, and procedures (UP2, UP3, PS2, PS1, and PS3). It was also emphasised that sticking to principles was regarded as ‘fundamental work’ (UP2). In their view, policies or decisions should be executed effectively after going through formal procedures (UP2 and UP3); playing by the rules could ‘simplify matters’ (UP2 and PS2), ‘avoid risks’ (UP2) and ‘protect the people in charge’ (PS2). They were mindful of their positions ‘at the macro and overall level’ (PS1) and their role of ‘taking the lead’ (UP3, PS2 and PS3) and ‘holding the direction’ (PS1 and UP2), as defined by the organisation (UP1, UP3, and PS3). They also stressed the significance of institutional analysis (UP2 and PS1) and discussed the fundamental missions of HEIs, such as talent development (UP1, UP2, and UP3) and public service (PS1). Other organisational issues they covered were ‘innovation’ (UP2 and UP3), ‘efficiency’ (UP1 and UP2), and ‘evaluation’ (UP2, UP3, and PS3).

Through the human resource lens, HEIs are considered an academic community where people are the most important resource. Most of the interviewees (UP1, UP2, PS1, and PS3) got close to teachers and students by, for example, visiting virtuous and respectable scholars in person (PS1), regularly visiting laboratories or faculties (UP1), and learning what was going on with students and young teachers, as well as how programmes were progressing through meetings with research teams (UP2). PS3 attended lectures and communicated with teachers and students when she was available. In addition, these leaders dealt with interpersonal relationships in a friendly manner (PS2), involved teachers in decision-making (UP3 and PS1), and empathised with, supported and empowered their subordinates and students (UP3, PS1, and PS2). Developing human resources is critical to a university’s long-term success. Participants thus recruited top talent and took supportive measures to train personnel (UP1 and UP3). As understood, a leader cannot play her role without people’s support and participation (PS1). The university should be a people-oriented organisation (UP2, UP3, and PS3), and the leaders’ role was to communicate (PS2), coordinate (PS2), collaborate (PS3), offer opportunities (PS2), and serve people (UP3 and PS1). Ultimately, they sought to create win–win situations, facilitating organisational development while also living up to most people’s expectations (UP2 and UP3).

Universities are generally like ‘a political jungle, alive and screaming’ (Baldridge, 1971, p. 21). Maintaining institutional legitimacy and gaining support from the CPC and the state became major concerns for Chinese institutional leaders. The interviewed leaders advocated the CPC’s leadership (PS1 and PS3) and sought the governments’ support via networking and diplomacy (UP1 and PS1). For example, they repeatedly cited President Xi Jinping’s political statements (PS1 and PS3) and speeches by the Minister of MoE (PS1) to justify their leadership behaviour. Externally, both the university president and the party secretary actively liaised with government agencies, recognising the importance of maintaining a positive relationship with their key stakeholders (UP1 and PS1). Internally, they built an alliance to achieve mutual success (UP1 and PS1). Interestingly, both PS1 and UP1 referred to their male counterparts as ‘brothers’. This is a clear example of alliance-building. They encouraged ‘frank communication’ (PS1) between each other and ‘put the university’s interest above everything else…not vying for the Alpha position’ (UP1). Despite their efforts to assemble a central coalition within HEIs, internal conflicts between the party secretary and the president seemed to exist (PS1, UP1, and UP3) or may occur (PS3). Nevertheless, they blamed disagreements on personal issues, claiming that the president and party secretary ‘did not implement the PRLPC well’ (PS3) and ‘did not collaborate well’ (PS1) if disputes occurred. The majority of the interviewees gave the PRLPC a favourable rating, such as ‘highly significant’ (PS1), ‘a very good governance system’ (PS1), ‘manifesting Chinese characteristics’ (UP2) and the ‘superiority of Chinese socialism’ (UP2 and PS3), ‘playing a good role in HEIs’ (UP2), and ‘ensuring that HEIs operate in the correct direction’ (UP1) and ‘ran smoothly’ (PS1). They also touched on and advocated the ‘Three Importance and One Largeness Decision-making System’ (PS1, UP1, and PS3) and the ‘CPC’s overall leadership on HEIs’ (PS1 and PS2), which were stated in a series of policy documents in the last decade. For instance, in 2011, the ‘Suggestions for Improving the Implementation of the Three Importance and One Largeness Decision-making System in the MoE-governed HEIs’ stipulated that all issues regarding Three Importance and One Largeness must be reported to the institutional leadership team for collective decision-making. Three Importance includes important strategic decisions, important leadership appointment and dismissal, and important institutional programmes, and One Largeness denotes the allocation of large funds. In 2018, strengthening political leadership was clearly included in the ‘Suggestions to the PCCPC in HEIs for Self-evaluation to Meet the Benchmark and for Competition to be First’. According to it, HEIs should adhere to and strengthen the CPC’s overall leadership and the PRLPC, advance all-round and strict governance of the CPC to the grassroots level, and implement the Three Importance and One Largeness Decision-making System.

Via the symbolic frame, institutional leaders see HEIs as cultural structures. As UP3 put it, ‘Culture is essential to any HEI. It is something that distinguishes this university from the rest’. To promote the image of U3, she started by developing various cultural products and renaming campus buildings. Afterwards, she held a ceremony introducing these cultural items and their backstories. Her cultural construction initiatives successfully instilled a sense of commitment, as she saw that some alumni were moved to tears. PS1, by contrast, used herself as a symbol. She learned about some anecdotes that had occurred in P1. Before assuming formal leadership, she convened a conference, announcing her intentions openly. With that speech, she effectively projected an image of integrity and justice, and ‘people were inspired’ (PS1).

In sum, no cognitive lenses were absent. Bolman and Deal’s four-frame leadership model can be used as a lens to gain a deep understanding of Chinese university leadership. The findings also imply that Chinese top-level institutional leaders use multiple tactics to fulfil their leadership roles.

### Relating leadership to the governance structure

As shown in Table 2, the structural framework was predominant among the interviewed leaders, suggesting a strong orientation towards managerial effectiveness. Such a phenomenon can be explained by the fact that leaders occupying top-level positions were more likely to emphasise fulfilling their positional roles.

**Table 2:** The frequency of codes in each frame

Association between leadership styles and leadership frames (Bolman & Deal)	Leadership styles	Managerial effectiveness	Managerial and leadership effectiveness	Leadership effectiveness	Leadership effectiveness
	Leadership frames	Structural frame	Human resource frame	Political frame	Symbolic frame
Interview analysis (coding frequency)	UP1	Very frequently	Occasionally	Frequently	Never
	UP2	Very frequently	Frequently	Occasionally	Never
	UP3	Very frequently	Frequently	Rarely	Occasionally
	PS1	Very frequently	Occasionally	Frequently	Rarely
	PS2	Very frequently	Very Frequently	Rarely	Never
	PS3	Very frequently	Occasionally	Frequently	Never

The table above was created using NVivo 12’s ‘hierarchy chart of nodes coded at this item’ which denoted the number of codes in each frame. To mark the frequency of codes in each frame for each transcript, we used five scales: Very frequently, Frequently, Occasionally, Rarely, and Never

Our data analysis indicates high managerial effectiveness but low leadership effectiveness, as the codes located in the structural frame are more frequent than those in the symbolic and political frames. Thus, at the risk of oversimplification, it can be inferred that Chinese university leaders tend to play a role of university managers more than institutional leaders. To better understand this key empirical finding, it is important to examine the distinctive institutional environment of Chinese higher education.

First, the state has played a dominant role in shaping higher education governance and university leadership. At the sector level, Chinese higher education features a ‘strong impact of an instrumental perspective in the formal governance arrangement’ (Dong et al., 2020, p. 835). HEIs are encouraged to implement government policies and the party’s agenda. As seen in the interviews, these university leaders frequently mentioned ‘policies’, ‘principles’, ‘rules’, and ‘regulations’. As a result of the strict leadership appointment and accountability scheme (Wang, 2010), top leaders are aware that they govern and manage HEIs on behalf of the state and the CPC and dare not break the rules. From this perspective, it is not difficult to understand why ‘playing by the rules could protect the people in charge’ (PS2). Since state involvement limits institutional autonomy and independence, Chinese university leaders can hardly become effective leaders as change agents driving organisational transformations.

Second, due to the hierarchical social values in China, management practices tend to be more authoritarian, often resulting in low trust in organisations (Wang & Clegg, 2002). Chinese HEIs have a hierarchical organisational structure. At the top are the CPCUC and the CUP. The CPCUC consists of the party (vice-) secretary and the (vice-) president, who are CPC members. Other internal governing bodies (e.g. the academic committee) are subordinated to the CPCUP. Below them are the teaching, learning, and research units. In such a structured and hierarchical organisation, fulfilling organisational roles, following the hierarchical chain of command, and playing by the rules may ‘simplify matters’ (UP2 and PS2) and ‘improve efficiency’ (UP1 and UP2); however, innovations within HEIs, which require leadership effectiveness, may be more challenging (Ma & Cai, 2021). More importantly, high power distance, respect for hierarchy, and expectation of obedience from subordinates to superiors have been established within the organisational culture. Consequently, leaders or managers are likely to rely more on rules and procedures than on their subordinates. This organisational culture with Chinese characteristics may be helpful in understanding why university leaders tend to be management-oriented rather than leadership-oriented; the latter requires trust (Joseph & Winston, 2005), while the former is characterised by adherence to rules.

Third, as opposed to the prevailing individualism in the West, China, among other Eastern Asian countries, values collectivism and thus places group harmony and social obligations over individual needs. To build a harmonious institutional environment, Chinese university leaders pay special attention to relationship-building with other leaders, subordinates, and stakeholders. As seen in Table 2, the human resource frame was the second most dominant frame in our study. Building relationships can be an effective way to build trust, and this is particularly important for collective decision-making in organisations with dual governance structures.

Regarding the governance arrangement, the PRLPC is enforced in a more intertwined manner than dualism. First, our study corroborates the overlap in personnel composition between the CPCUC and the CUP (Liu, 2017; Wang, 2010). According to the Higher Education Law, the CUP is the highest institutional governing body for teaching, research, and other administrative affairs (Article 41). However, in three sampled HEIs (U1, U2 and U3), all the administrative and party leaders attended both the CPCUC and the CUP for collective decision-making. Since the issues to be discussed and decided upon can be both, it is difficult to separate academic from political administration. Second, rather than differentiating academic leaders from politicians, these institutional leaders can be more precisely defined as academic politicians. As noted by PS3, ‘university leaders should be politicians and educators. Both are indispensable.’ Actually, being socialist politicians as well as educators is the unified requirement for the entire university leadership team, according to President Xi Jinping’s speech (Xi, 2019) and the ‘Issuing the Opinions on Sustaining and Improving the PRLPC in Regular HEIs’, released in 2014. Furthermore, it should be noted that the interviewees’ trajectories proved no distinct demarcation in leadership appointment between the dual tracks.

Under this governance umbrella, the leadership roles of party secretaries and university presidents do not differ significantly. The interviewees’ narratives about leadership experiences demonstrate that party secretaries sometimes manage teaching, academic, and student affairs (PS1, PS2, and PS3), and university presidents are also involved in political construction (UP1 and UP3). In addition, most of them even supervise students or conduct research (PS1, UP1, PS3, UP2, and UP3). Furthermore, the scope of authority is not clearly distributed between party secretaries and presidents. As noted by PS1, ‘the party secretary and the president manage the university side by side. Sometimes, the president takes charge of execution and implementation. Sometimes they are the party secretary’s business’. This vague ‘dualism’ can easily give rise to internal conflicts between the two heads; however, none of the interviewees ascribed ‘internal conflicts’ to the governance structure. Even so, ‘the relationship between the secretary and the president can be sensitive’ (Liu, 2017, p. 274) because the two key positions are endowed with ‘both political and administrative authorities over university affairs’ (Han & Xu, 2019, p. 937).

Nevertheless, the party secretary’s leadership position may be higher than the president’s, especially in the last decade. In the six sampled universities, the party secretary chaired the CPCUC, while the president served as the party vice-secretary. Some ideas shared in the interviews imply a positional hierarchy. For example, UP1 said that the party secretary and the president were ‘not vying for the Alpha position’, and UP3 said that ‘the party secretary is actually the big wig’. Furthermore, by centralising final-decision authority at the top, the policies on the ‘CPC’s overall leadership on HEIs’ and the ‘Three Importance and One Largeness Decision-making System’ have aided in strengthening the authority of political leaders, particularly the party secretary. At the same time, administrative leaders’ authority, especially that of the president, has diminished, as some of the authority that should have been theirs has been distributed among CPCUC members. Regardless of the power hierarchy, the two kinds of leaders worked collaboratively with each other. For example, before each round of CPCUC meetings, the party secretary initiated private conversations with the president about matters to be discussed (UP1 and PS1), and she could set the agenda. For the items that the secretary and the president could not agree on, they continued to negotiate. Until both sides reached a consensus, the party secretary reported the issues to the CPCUC meetings for group discussion and final decisions (PS1).

## Theoretical foundations and empirical realities—a critical reflection on China’s complex university leadership

This article sets out to address two research challenges: how to provide valid analytical interpretations of China’s complex university leadership while also responding to the theoretical foundations that may help us to shed light on this complex institutional governance arrangement. The four frames offered in Bolman and Deal’s (2013) framework could be said to offer considerable face validity, as our interviewed HEI leaders indeed perceived their leadership activities along all four cognitive frames. However, we also found that the structural frame was clearly dominant, whereas the symbolic frame was the least used. This finding is in stark contrast to studies focusing on American and Canadian higher education, where the symbolic frame was more evident than the structural frame (Lavigne & Sá, 2021; Welch, 2002). However, by utilising the distinction between leadership and management effectiveness, we found that Chinese HEI leaders end up in roles more associated with university management than institutional leadership, which hints at why the structural frame is so dominant. Another interesting finding is that no significant differences can be spotted between party secretaries and university presidents regarding their leadership frames, which contrasts with previous studies (Jiang & Li, 2012; Jiang et al., 2008; Ling & Xu, 2020). There are several possible explanations for this. First, their daily work along political and administrative lines may be closely intertwined due to the dominant role of the state in the governance of universities (Dong et al., 2020). Second, the top leaders may be quite mobile in their careers—switching between the party secretary and president roles—aligning their views of leadership responsibilities over time. Here, our empirical data points to a dynamic that static cognitive frameworks on leadership tend to struggle with: how leadership experience transforms over time, blurring the categories and distinctiveness of theoretical leadership models.

The different dimensions offered by the Bolman and Deal (2013) framework were nevertheless useful in helping elucidate the mechanism underlying the phenomenon of ‘dancing in a cage’ (Yang et al., 2007), a metaphor used to describe Chinese university governance in which ‘academic freedom has always been viewed as problematic in the country’ (Zha & Shen, 2018, p. 447). Based on our analysis, we argue that the structural frame dominates the cognitive frames of Chinese university leaders, leading to the construction of (‘iron’) ‘cages’ in leadership perceptions and behaviour. However, achieving the high degree of bureaucratic control implied by the dominant structural frame may come at the expense of academic development. While China has endeavoured to build world-class universities, its dual leadership model could be a hurdle that restrains creativity and boldness.

Our empirical analysis also provides evidence that the various dimensions and categories of leadership perceptions and behaviour often found in cognitive leadership frameworks are not as mutually exclusive as presented and need to be related to the specific empirical context of their application. In our study, there seems to be considerable overlap between the structural and political frames. This is probably because Bolman and Deal distinguish administration from politics; however, this distinction is less meaningful in a Chinese context where political control and university administration are integrated (Huang, 2017). As university leadership is increasingly faced with strengthened accountability claims globally, driving the establishment of larger management teams at the top level (Meyer et al., 2007), future studies should take into account the tight personal networks surrounding top leaders—in China and elsewhere—and how these might affect leadership perceptions and practices.

Our study also exposes another weakness of cognitive theories of leadership. It reminds us that understanding university leadership cannot leave aside the institutional environment—the political sphere and the governance arrangement in which the leadership is embedded. Institutional theorists have distinguished between organisational and institutional leaders (Washington et al., 2008, p. 720). The former prefer to achieve objectives instrumentally, while the latter emphasise core values and norms in guiding organisational practices. One could argue that in the contexts where politics and administration are intertwined, cognitive and normative dimensions of the leadership function (Meyer et al., 2007) should be more clearly distinguished. Thus, cognitive leadership theories could, in general, benefit from incorporating the institutional perspective to better understand the embedding of Chinese HEI leaders in the dual governance structure.

## Conclusion

As this was an in-depth study of selected institutional leaders with a small sample size, our study has clear limitations in terms of making bold empirical generalisations. Nonetheless, our study makes several contributions to the literature on the dual leadership model in China. We have indicated how leadership perceptions seem to favour structural frames of interpretations above symbolic ones. We suggested interpretations for the lack of differences between party secretaries and presidents in their leadership perceptions and practices, and we proposed a mechanism for why Chinese HEI leaders are ‘dancing in a cage’ with respect to their leadership discretion. In conclusion, we would also argue that future explorations of university leadership in China should incorporate more contextual frameworks, such as institutional perspectives that focus on the role of environmental norms and values in shaping management and leadership behaviours (Meyer et al., 2007). Such a perspective would also be in line with the suggestions by Marginson and Yang (2022) to comprehend Chinese higher education based on pluralistic cultural identities and trans-positionality. Indeed, the Chinese model of higher education is a mix of ‘Western and Chinese (mainly Confucian) elements’ (Zha et al., 2016, p. 273) as well as strong state control. In such a context with a plurality of values, some newly established universities have become arenas where presidents and party secretaries have acted more in line with the characteristics of institutional leadership (Ma & Cai, 2021). Thus, in future theorising on Chinese university leadership, a pluralist cultural perspective should be taken.

## Supplementary Information

Below is the link to the electronic supplementary material.

Supplementary file1 (DOCX 19 KB)

Supplementary file2 (DOCX 18 KB)
