# Supplementary material for: University managers or institutional leaders? An exploration of top-level leadership in Chinese universities
Source: High Educ (Dordr). 2023 Apr 10:1–17. Online ahead of print. doi: 10.1007/s10734-023-01031-x (PMC10088719; doi:10.1007/s10734-023-01031-x)
Supplement: Supplementary file 1 — Supplementary file1 (DOCX 19 KB) [file 10734_2023_1031_MOESM1_ESM.docx]

## Appendix 1: Interview Guide

Note: The interview guide is provided as the Appendix to our article. Since we only published the findings on leadership and governance, we merely presented the interview questions relevant to the data reported. In each interview, the participants answered more questions than those in the Appendix.

1. How do you exercise leadership in your current leadership position? What achievements have you got and what difficulties have you faced?

2. What ideas and principles do you adhere to?

3. What kind of leadership styles have you developed? Or can you use three to six keywords to describe yourself as a university leader?

4. What factors influence your above-mentioned leadership styles?

5. What areas do you attach importance to for the university development?

6. Are you also involved in or responsible for research and teaching issues? Do teaching and research support or contradict university management according to your own experiences?

7. Can you provide an example of your typical workday and your typical rest day in terms of time and task distribution?

8. Can you give opinions on university leaders’ roles and responsibilities, as well as university governance and management?
