# Supplementary material for: University managers or institutional leaders? An exploration of top-level leadership in Chinese universities
Source: High Educ (Dordr). 2023 Apr 10:1–17. Online ahead of print. doi: 10.1007/s10734-023-01031-x (PMC10088719; doi:10.1007/s10734-023-01031-x)
Supplement: Supplementary file 2 — Supplementary file2 (DOCX 18 KB) [file 10734_2023_1031_MOESM2_ESM.docx]

## Appendix 2: The coding table generated from this study

| **Frame** | **Frame-Related Actions** | **Frame-Related Issues** |
| --- | --- | --- |
| Structural | Adding new structural units; Budgeting; Defining clear goals; Developing new information (reform & innovation); Distributing responsibilities according to organizational structure; Planning processes; Evaluating results; Identifying principal problems; Learning and implementing policies; Making and following rules & regulations; Obeying the law; Restructuring/reorganizing; Supervising implementation | Evaluation; Clarity of goals, roles, positions and direction; Development of new information (innovation); Discussions of institutionalization; Efficiency; Institutional analysis; Implementation and execution; References to policies, rules, principles and procedures |
| Human Resource | Dealing with interpersonal relations; Getting close to teachers and students; Involvement in decision-making; Recruiting and training personnel; Empathizing with, supporting and empowering others | Discussions of people-oriented organization; Emphasis on win-win; References to communication, coordination, collaboration and empowerment; References to servant leadership (leading is serving); References to the importance of participation |
| Political | Political advocacy; Building alliances; Networking with the key stakeholder | References to government support; References to political leadership; References to internal conflicts |
| Symbolic | Creating ceremonies; Using herself as a symbol; Developing cultural symbols | Discussions of institutional culture and its symbolic importance |

Source: Adapted from Bolman and Deal (1991)
